# A Review of the Present Knowledge of Trench Fever

**Published:** 1917-12

**Authors:** 


### TRENCH FEVER

***A Review of the Present Knowledge of Trench Fever.*** Compiled from the Articles Listed (p. 136).

Fever known among the British troops as P. U. O. (pyrexia of undetermined origin) has greatly increased in the British troops in France during the past year. Under this term, which it is admitted is an unfortunate one, have been included through error of diagnosis cases of paratyphoid fever, of influenza, and especially of trench fever.

During the latter part of the year 1915 and in 1916, in Northern France, a disease known as trench fever became gradually recognized as a distinct, specific infection. Cases of this disease were also reported from the armies in Salonica and Mesopotamia, and apparently a similar affection occurred among the German troops. In some of the armies in Northern France trench fever has caused almost one-third of all the sickness. In one division during a year, a monthly average of about 350 cases of this affection were sent to field hospitals. Macgregor and Ramsay have given evidence of individuals who have contracted the disease in England. The disease has occurred particularly in France in those who have been in the trenches or among those who have cared for the sick in the hospitals.

*Clinical features.* — The incubation period of trench fever as determined experimentally varies from six to twenty-two days. The disease appears particularly in two clinical types, one in which there is a short, evanescent fever lasting from a few days to a week, and frequently followed after a few days of apyrexis by a single short relapse; and a second in which there is a series of relapses of the fever interrupted by periods of apyrexia. In both forms as a rule the onset is sudden and the affection is characterized by headache, dizziness, pain in the back and particularly in the legs, constipation, and a sharp rise of temperature, usually to 102 or 103 degrees Fahrenheit. Pain in the shins or muscles of the legs is a very common complaint. It is often very distressing and renders the patient restless at night. The pain in the legs occurs particularly at the points of insertion of the muscles to the bone, where apparently an actual inflammatory process takes places. The pain does not affect the joints. The face is usually flushed but the eyes are clear and bright. The conjunctivae are not suffused as in Dengue fever. The tongue is usually coated and there is anorexia. The pulse is usually in the neighborhood of 100 and the heart is normal in the early stages of the disease. Pulmonary catarrh is absent. There is no albuminuria and no enlargement of the spleen. His, and later Zollenkopf, also reported under the name of "Volhynian fever" the occurrence of a somewhat similar disease in the German troops. However, His states that the spleen is almost always enlarged; and Zollenkopf, that it is usually increased in size and that a rash is present. Jungmann concluded that Volhynian fever was a form of typhus.

In the first form mentioned, after about the third to the sixth day of fever, there is often a drop in the temperature and the temperature remains in the neighborhood of normal for several days. Then a short relapse of fever, lasting only a day or two, takes place. In the second type there may be several attacks of fever lasting three or four days or longer, in which the temperature may reach as high as 104 or 105 degrees Fahrenheit. Between the attacks of fever there are periods of apyrexia of about the same length of time. The periods of fever and relapse, however, may vary; for example, in three cases reported by Herringham the fever lasted 7, 10, and 13 days respectively, and the periods of apyrexia 5, 8, and 11 days. Houston and Mc Cloy have also described what they call a myalgic type in which there is one period of fever lasting from a few days to a week. After this the temperature falls to normal, but the severe pain in the legs continues. Occasionally a sudden rise of the temperature during this period occurs. They have also described a septicemic type in which the enterococcus was isolated from the blood. Other observers have not reported upon the septicemic type. Albuminuria has usually been reported as not present. Mandel however, states that albumin is frequently present and that in severe cases hyaline and coarsely granular casts and red blood cells are present, the casts frequently persisting after the albumin has disappeared. Possibly these cases are related to " Trench Nephritis. "

*The blood* shows a slight relative increase in the large and small lymphocytes and a reduction in the hemoglobin. A punctate basophilia of the red blood corpuscles is present.

*The prognosis* in very good but the condition in the severe form is apt to be followed by a certain amount of debility. The most important sequelae are anemia which occurs in 10 o/o to 15 o/o of the cases, and disordered action of the heart. The latter is very common after the severe cases and in these tachycardia is also frequent.

*The diagnosis* is often difficult. The pain in the legs in the neighborhood of the shins is the most constant symptom. The disease may be distinguished from relapsing fever by the absence of the Spirillum recurrenti from the blood. From influenza it is particularly differentiated by the absence of any catarrhal symptoms and by the lack of prostration in trench fever early in the disease. In Dengue fever a rash is usually present and the distribution of pain is different. Trench fever may be distinguished from malaria, undulant fever, and enteric infections by the appropriate blood and serum examinations.

*The treatment* is entirely symptomatic. Aspirin and morphine relieve the pain, but seem to exert no influence over the course of the disease. Quinine and salicylic acid and intravenous injections of antimony, salvarsan, and eusol did not affect the fever. The injection of the serum of convalescents in several instances also has not appeared to shorten the attacks.

The infectious nature of trench fever was demonstrated by McNee, Renshaw, and Brunt, by the transmission of it to healthy men by the subcutaneous injection of blood taken from patients during the active stage of the disease. In this way it was determined that the shortest incubation period was six days and the longest twenty-two. It was not possible to produce the infection with the blood serum of infected individuals unless hemolysis of the red blood cells had taken place. The plasma was also not found to be infective unless hemolysis had occurred and filtered serum and plasma were also inactive. The red blood corpuscles, however, were found to be capable of causing the disease even after being washed several times in saline solution. The infected agent is not apparently filterable : if the red cells from the patient are ground up with fine sand and the blood then filtered, the filtrate is quite inactive.

*Transmission.* — It is commonly believed that transmission of this infection occurs through the louse. Muir, however, opposed this view and stated that in a small percentage of the cases the absence of lice was certain. Davies and Weedon report one case in which one of them was twice bitten by a louse, the first time just after it had been allowed to feed upon a case of the disease and a second time twenty-four hours later. A typical attack of trench fever developed twelve days later in the individual so bitten. Hurst also reports that Captain Urquhart developed the short form of trench fever after allowing lice taken from a patient with this form of the disease to bite him. He also gives some evidence of a soldier who was infected with vermin and who shortly afterwards contracted the disease.

*Etiology.* — Extensive investigations regarding the etiology of the disease have been made, but the organism causing it has apparently not yet been definitely detected by the microscope or the ultra microscope either in fresh or stained preparations. Sergent, and later Renaux, found semi-lunar bodies in the blood which occurred particularly from two to four hours following each relapse. Houston and McCloy regarded the enterococcus which was isolated from the urine in the majority of their cases as the cause of the infection. Dimond has described a protozoon, a hemogregarine, in twelve cases, both in the venous blood and in material obtained by splenic puncture. He states the organism was only discovered after the blood had been drawn in 1.8 o/o citrate solution and then hemolysed by the addition of a .5 o/o solution of saponin. The parasite was found after this solution had been centrifuged. This discovery, however, has not been confirmed. Pappenheimer found discoid bodies in blood films and in specimens taken from near the periosteum, and in cultures from the blood and tissues of trench fever patients. He later concluded that these were not of a parasitic nature. Patterson has very recently found spirochaetae in the urine in fifteen cases of trench fever. Nankiwell and Sundell have also found a spirochaeta in twelve of fifteen typical cases of trench fever. No work was done regarding the pathogenicity of this organism for man or animals. In eighty control cases, either healthy or suffering with other diseases, spirochaetae were not found in the urine. These authors did not succeed in finding the spirochaeta in the blood in trench fever cases or in cultivating it from the blood stream, as, according to them, Reimer claims to have done. However, Stoddard has also just reported the presence of spirochaetae in 33 o/o of individuals in the examination of specimens taken from the urethra or the urine. Hence the mere presence of a spirochaeta in the urine in trench fever cases can not be regarded as conclusive evidence that this organism is the cause of the disease or that it bears any direct relation to it.

In relation to prevention, it appears that at the present time it is most important to determine definitely whether the disease is transmitted through the medium of the louse. This should not be difficult to demonstrate. The parasite causing yellow fever is not yet discovered but the disease is prevented and its spread controlled by the knowledge of its method of transmission by the mosquito.

R. P. S.

REFERENCES

1. Graham. *The Lancet.* Sept. 25, 1915.
2. Hunt and Rankin. *The Lancet,* Now. 20, 1915.
3. Mc Nee, Brunt, and Renshaw. *British Medical Journal,* Feb. 12, 1916.
4. His, *Berl. Klin. Woch.,* July 3, 1916.
5. Zollenkop. *Deut. Med. Woch.,* Aug. 24, 1916.
6. Hurst. *The Lancet,* Oct. 7, 1916.
7. Muir. *British Medical Journal,* Nov. 11, 1916.
8. Davies and Weedon, *The Lancet,* Feb. 3, 1917.
9. Coombs. *The Lancet,* Feb. 3, 1917.
10. Mc Gregor. *British Medical Journal.* Feb. 17, 1917.
11. Ronsay. *British Medical Journal,* Feb. 17, 1917.
12. Jungmann and Kweyzinski. *Deut. Med. Woch.,* March 22, 1917.
13. Renaux. *Comptes rendus de la Société de Biologie,* April 21, 1917.
14. Herringham, *British Medical Journal,* June 23, 1917.
15. Renaux. *Hygiène Publique Internat'onale,* June 1917, p. 808.
16. Jungmann. *Bulletin de l'Institut Pasteur,* 1917, p. 453.
17. Grierson. *British Medical Journal,* 1917.
18. Dimond. *The Lancet,* Sept. 8, 1917.
19. Patterson. *British Medical Journal,* Sept. 29, 1917.
20. Stoddard. *British Medical Journal,* Sept. 29, 1917.
21. Pappenheimer, Vermilye, Mueller. *British Medical Journal,* Oct. 13, 1917.
22. Pappenheimer. *British Medical Journal, Oct.*27, 1917.
23. Nankivell and Sundell. *The Lancet,* Nov. 3, 1917.
24. Rutherfurd. *British Medical Journal,* Sept. 16, 1916.
25. Wright. *British Medical Journal,* July 27, 1916.
26. Dudgeon, *Lancet,* Dec. 1, 1917.
27. Nankivell and Sundell, *Lancet,* Dec. 1917.
28. Moreland, Mc Crea and Dickson. *Lancet,* 1917, p. 796.

*Le Gérant :* O. Porée. Paris — imp. Lahure, 9, rue de Fleurus.

